# Individual and COVID-19-Specific Indicators of Compliance with Mask Use and Social Distancing: The Importance of Norms, Perceived Effectiveness, and State Response

**DOI:** 10.3390/ijerph18168715

**Published:** 2021-08-18

**Authors:** Jordan A. Gette, Angela K. Stevens, Andrew K. Littlefield, Kerri L. Hayes, Helene R. White, Kristina M. Jackson

**Affiliations:** 1 Department of Psychological Sciences, College of Arts and Sciences, Texas Tech University, Lubbock, TX 79410, USA; andrew.littlefield@ttu.edu; 2 Department of Behavioral and Social Sciences, Center for Alcohol and Addiction Studies, Brown University, Providence, RI 02912, USA; angela_stevens@brown.edu (A.K.S.); Kristina_Jackson@brown.edu (K.M.J.); 3 American Academy of Addiction Psychiatry, Providence, RI 02914, USA; kerri@aaap.org; 4 Center of Alcohol Studies, Sociology Department, Rutgers University, Piscataway, NJ 08854, USA; hewhite@smithers.rutgers.edu

**Keywords:** COVID-19, compliance, norms, perceived effectiveness, government response

## Abstract

COVID-19 is a global pandemic that has resulted in widespread negative outcomes. Face masks and social distancing have been used to minimize its spread. Understanding who will engage in protective behaviors is crucial for continued response to the pandemic. We aimed to evaluate factors that are indicative of mask use and social distancing among current and former college students prior to vaccine access. Participants (N = 490; 67% female; 60% White) were current and former U.S. undergraduate college students. Perceived effectiveness and descriptive norms regarding COVID-19 safety measures, COVID-19-related news watching and seeking, state response timing to stay-at-home mandates, impulsivity-like traits, affect (mood), and demographic variables were assessed. Results found that greater perceived effectiveness indicated increased personal compliance within and across behaviors. Greater norms related to compliance within behaviors (e.g., indoor norms related to indoor compliance). Increased perceived stress, anxiety, and negative affect indicated greater compliance. More positive affect was associated with less compliance. Being non-White, compared to White (*p* < 0.001), and female, compared to male (*p* < 0.001), were associated with greater compliance. Overall, early implementation of stay-at-home orders, exposure to COVID-19-related news, and increased perceived effectiveness are crucial for health safety behavior compliance. Findings are important for informing response to health crises, including COVID-19.

## 1. Introduction

COVID-19 has been identified as a global pandemic. At the time of this writing, the United States alone has recorded approximately 34 million total cases and more than 600,000 deaths. Importantly, young adults have played a major role in the spread of COVID-19 [1,2]. Further, there is a positive relation among compliance with safety measures, such as social distancing and sanitation behaviors, and age, suggesting younger individuals are less likely to comply [3]. Many health experts, including the Center of Disease Control [4,5], have stated that the use of face masks and engaging in social distancing (i.e., maintaining at least six feet of space between individuals) can help slow the spread of the virus. Further, compliance with one safety behavior (e.g., mask use) increases the odds of compliance with additional safety behaviors (e.g., hand washing [6]). It is important to understand the long-term effects of the timing of state-level orders and whether delayed orders impact individual mask use and social distancing to inform the currently handling of this global pandemic as well as future pandemics [7]. As such, identifying indicators of mask usage and social distancing behaviors, particularly among young adults, could serve to assist communities in effectively promoting health safety behaviors and reducing spread of COVID-19.

### 1.1. COVID-19-Specific Predictors of Compliance

Several cognitive and contextual factors including norms, perceived effectiveness, and exposure to COVID-19-related information are likely linked with safety behavior compliance. Descriptive norms refer to an individual’s beliefs about others’ behavior and have been studied in the context of numerous health behaviors including vaccinations for influenza [7], human papilloma virus [8], and substance use [9,10]. Looking specifically at COVID-19, Abdallah and Lee [11] found a positive relation between peer descriptive norms regarding intent to get vaccinated and personal intent to receive a COVID-19 vaccine. Further, individuals’ attitudes regarding a behavior influence their engagement. Like descriptive norms, this pattern of attitudes influencing behavior has been seen across a myriad of health behaviors such as alcohol use [12] and diet [13]. Regarding COVID-19, Vally [14] found that, in the United Arab Emirates (UAE), the perceived efficacy of COVID-19 health behaviors (e.g., social distancing, disinfection) was positively related to increased behavior engagement. Further, the more important that U.S. college students perceived the vaccine to be (i.e., positive attitudes), the greater their intentions were to receive a vaccine [11]. Thus, it is likely that more positive attitudes regarding the effectiveness of mask use and social distancing (e.g., perceived effectiveness) relate to increased adherence to these guidelines.

In addition to norms and attitudes, increased exposure to information regarding COVID-19 and relevant safety precautions could also serve to increase mask use and social distancing compliance. As individuals are presented with information demonstrating the effectiveness of CDC guidelines and increasing rates of the virus, their likelihood of compliance may increase as a result of increased awareness. For example, in the UAE, those who read the government public health guidelines, compared to those who had not, were more likely to comply [14].

Another contextual factor worth considering is individual states’ responses to COVID-19, which reflect the cultural norms surrounding the pandemic. As COVID-19 first began to spread at the beginning of 2020, individual states within the U.S. implemented mandates and advisories at discrepant times. Stay-at-home mandates and advisories first began on 17 March 2020, with 43 states and territories implementing either a mandate or advisory by the end of March 2020. However, other states demonstrated a more delayed response with seven states implementing stay-at-home mandates or advisories in April or later, and an additional six states and territories never implementing a stay-at-home order in 2020 [15]. Importantly, research has shown that state-level COVID-19 safety measures (e.g., stay-at-home orders) helped to slow the spread of the pandemic [16,17] and that the early implementation of stay-at-home mandates may evoke additional compliance such as mask usage. In fact, compared to residents of New York City and Los Angeles, two cities with early stay-at-home order implementation, a broader sample of U.S. citizens were less likely to report always wearing masks in public and engaging in stay-at-home behaviors [18]. It is important to understand the long-term effects of the timing of state-level orders and whether delayed orders impact individual mask use and social distancing to inform the current handling of this global pandemic as well as future pandemics [19].

### 1.2. Additional Predictors of Compliance

There is evidence to suggest that individual traits and affect (i.e., mood) may influence adherence to COVID-19 safety behaviors. Notably, several studies have reported increased rates of anxiety and stress following COVID-19 [20] and these increases in negative affect have been linked to a greater initiation of COVID-19 safety compliance such as hand washing and social distancing [20,21]. With regard to COVID-19 safety compliance, it could be that individuals who are experiencing increased anxiety and stress as a result of the pandemic are more inclined to follow CDC guidelines as a means of mitigating these negative affective states. Conversely, Pollack et al. found that greater psychological distress was related to decreased compliance in a sample of Israeli adults [22]. Given the cross-sectional nature of this study, however, it could be that the inability to engage in safety behaviors, such as staying at home due to work demands, could be influencing stress. Further, Pollack et al.’s data were collected between 28 March and April 2020 during the outset of the pandemic when less was known about the effectiveness of different safety behaviors, which could have impacted engagement in these behaviors. Additionally, extant work has focused on negative affect, but it is important to know how positive affect may influence compliance.

In addition to affect, trait impulsivity may also be related to COVID-19 compliance, as suggested by the following studies. In line with Jessor’s Problem Behavior Theory [23,24], those higher in impulsivity may be less likely to comply with safety behaviors as this theory argues that there is a subset of individuals who are more likely to engage in risk behaviors, and this pattern has been seen in the extant literature. For example, “low self-control” (i.e., a composite of items related to impulsivity, risk-seeking, and related traits) at age 20 predicted decreased compliance with COVID-19 safety precautions at age 22 after controlling for sex, socioeconomic status, education, and migrant status [25]. Additionally, greater attention-deficit hyperactivity symptomology, which can manifest as increased impulsivity [26], and risk-taking behavior were also associated with lower adherence to COVID-19 safety behaviors [22]. Though these findings suggest that impulsivity may be related to compliance, impulsivity is a multifaceted construct [27] and extant literature has yet to examine whether different facets of impulsivity are related to COVID-19 safety behaviors. Following personality theories, research has established five distinct but related facets of impulsivity: (1) positive urgency relates to acting upon positive emotional impulses, (2) negative urgency relates to acting upon negative emotional impulses, (3) sensation seeking refers to a tendency to seek out new and risky experiences, (4) lack of perseverance refers to the inability to sustain attention on tasks, and (5) lack of premeditation/planning refers to consideration of consequences prior to acting on impulses [28]. It is reasonable to hypothesize that different domains of impulsivity may exhibit differential relations with safety behavior compliance. In particular, higher sensation seeking may be especially indicative of noncompliance, as individuals higher in sensation seeking may seek out riskier experiences (e.g., risky driving [29]).

Demographic variables may also influence whether individuals engage in COVID-19 safety behaviors. Reports by the CDC have found that racial minorities are at increased risk of a COVID-19 diagnosis and have higher mortality rates from the virus [30]. These discrepancies are the result of several factors including contextual and systemic issues (e.g., access to healthcare), but to date, only a few studies have examined how rates of safety behavior engagement vary by racial identity. For example, Block et al. [31] found that 90% and 83% of Black respondents endorsed complying with social distancing and mask use when outdoors always or most of the time, respectively, but there was no comparison group in this study. A CDC report found that, in April 2020, White individuals endorsed less mask use than non-White individuals, but rates of self-reported mask use became comparable for all racial groups by May 2020 [32]. Individuals identifying as a racial minority may be more likely to comply with safety recommendations in response to the increased prevalence of COVID-19 and higher mortality rates among racial minorities. Notably, Czeisler et al. [18] found that White individuals were less likely to support stay-at-home orders compared to all other racial groups. However, there were no racial differences in social distancing or public mask use. Conversely, Rader et al. found that White individuals reported lower intention to use a face mask than all other racial groups [33]. Overall, given the mixed findings regarding the role of race in compliance, further examination of potential differences is warranted. In addition to race, biological sex has been implicated in the adherence to pandemic safety behaviors. It appears that males, compared to females, are less likely to comply with safety behaviors such as mask use, hand washing, sanitization, and social distancing, even after controlling for variables such as work environment [34]. These findings are consistent across several countries including the United States [18], France [3], Switzerland [25], Israel [22], and multinational samples (i.e., the United States, the United Kingdom, Italy, China, Japan, and Korea [35]).

### 1.3. The Current Study

The purpose of the present study was to identify demographic and COVID-19-related contextual factors implicated in individuals’ adherence to COVID-19 safety behaviors, namely indoor and outdoor mask use and social distancing, among current and former undergraduate college students. A number of individual traits and affective variables were also examined to determine if previous findings regarding these variables are maintained in a large multi-site sample comprising U.S. current and former college students. With regard to the overarching study aim, the following hypotheses were proffered:

1. Greater perceived norms for compliance and more favorable attitudes (i.e., greater perceived effectiveness) regarding safety behaviors would be indicative of greater personal compliance [12].
2. Greater exposure to COVID-19-related news would be related to increased compliance due to potentially heightened awareness of the effectiveness of these behaviors [15].
3. Compliance would be greater for individuals in states that enacted stay-at-home orders earlier [18].
4. Females would be more compliant with safety behaviors than males [18].
5. White individuals would be less compliant with safety behaviors compared to non-White individuals [18,31].
6. Greater levels of negative affect and anxiety would be related to increased compliance [20].
7. Relations involving positive affect are exploratory, as extant literature has primarily focused on negative affect [20,21,22].
8. Broadly, each impulsivity facet would be related to decreased compliance, with sensation seeking (i.e., the tendency to seek out new and risky experiences [28]) demonstrating the largest effect sizes.

## 2. Materials and Methods

### 2.1. Participants

**Figure 1:**
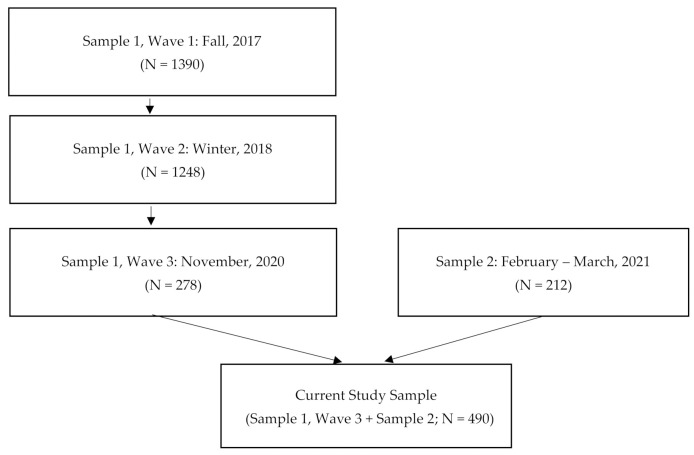
Flowchart of participant recruitment for the present study. The sample for the current study was derived from two independent samples. Data from Sample 1 were collected in three waves. Sample 1, Wave 1 was collected in the fall of 2017; Sample 1, Wave 2 was collected in the winter of 2018; and Sample 1, Wave 3 was collected in the fall of 2020. For Sample 1, demographic data were collected at Wave 1 and impulsivity data were collected at Wave 2. All COVID-19-related variables were collected at Wave 3. Data for Sample 2 were collected at a single timepoint between January and February 2021. The total sample size (N = 490) for the current study is comprised of Sample 1, Wave 3 plus Sample 2).

Participants were recruited for this study in two parts. Participants for Sample 1 were originally recruited from three U.S. state universities (two in the Northeast and one in the Northwest) as part of a larger study on college student substance use (see White et al., [36] for further details); they were aged 18–24 and endorsed past-year alcohol and cannabis use. Online surveys were completed in the fall of 2017 (Wave 1) and winter of 2018 (Wave 2). A subset of original study participants, who were first-year and sophomore students at baseline, were recontacted to participate in a series of surveys on COVID-19, the last of which was completed online in November 2020 (Wave 3; *n* = 278). For the present analyses, data were primarily derived from Wave 3, though demographic variables were collected at Wave 1 and impulsivity variables were collected at Wave 2, because they were not administered at Wave 1 to reduce participant burden. These participants received a USD 25 gift card for their participation in Wave 3. All participants were enrolled as undergraduates in a university during the Wave 1 data collection. However, some students were no longer enrolled at Wave 3. All procedures were approved by the coordinating university’s Institutional Review Board (IRB). See Figure 1 for a flowchart of participant recruitment for each sample.

Sample 2 participants (N = 212) were recruited through undergraduate psychology courses at a large southwestern university in January and February 2021 and these individuals received course credit for their participation. This second sample was recruited to increase national representation in the sample, specifically to increase variability in state response timing. 

The overall sample (N = 490; 66.7% female) had a mean age of 21.95 years (SD = 2.29, range = 19–27) and was predominately non-Hispanic White (59.6%) followed by White-Hispanic (10.5%), non-Hispanic Asian (9.5%), non-Hispanic multiracial (6.2%), Hispanic-other (4.6%), non-Hispanic Black (3.7%), Hispanic multiracial (1.2%), non-Hispanic American Indian (10.0%), Hispanic Black (0.8%), Hispanic Asian (0.8%), Hispanic American Indian (0.6%), non-Hispanic Indian (0.4%), and 2.1% identified as “other.” In the full sample, students reported residing in 24 different states at the time of survey completion; 56.9% of participants resided in states that implemented an early stay-at-home order (i.e., prior to 1 April 2020). At the time of data collection for both samples, CDC guidelines broadly recommended use of social distancing and face masks in all indoor settings outside of the home as well as outdoor settings in which social distancing was not feasible. In addition, at the time of survey administration for Sample 1, COVID-19 vaccinations were not yet available in the U.S., and for Sample 2, they were only available for healthcare workers and those with high-risk health conditions.

For both samples, all participants were aged 18 or older and provided consent to participate. Data were de-identified with participant IDs used to link data across waves for Sample 1; Sample 2 responses were collected anonymously. All procedures were approved by the corresponding universities’ IRBs and a “Certificate of Confidentiality” was obtained from the U.S. National Institute on Drug Abuse for Sample 1.

### 2.2. Measures

All measures were completed via an online survey software. In addition to the measures included in the present study (described below), additional information was collected regarding substance use (e.g., use patterns, motives, consequences), contextual factors (e.g., living environment, extracurricular activity involvement), social media usage, and additional COVID-19-related indicators (e.g., disruptions to education caused by COVID-19). Given the novelty of COVID-19 and limited extant literature for the present analyses, we selected variables that have historically been demonstrated to relate to health behaviors (e.g., norms, affect, impulsivity) as well as measures from The CoRonavIruS Health Impact Survey (CRISIS) V0.3: Adult Self-Report Form: National Institute of Mental Health [37]. Available from: https://www.nlm.nih.gov/dr2/CRISIS_Adult_Self-Report_Follow_Up_Current_Form_V0.3.pdf (accessed on 1 December 2020). Additionally, we aimed to include indicators that may be more specific to COVID-19, such as access to COVID-19-related news, which could inform safety behavior decision making. Social distancing and mask use variables were selected, as these health behaviors were more specific to the COVID-19 response recommendations compared to more broad health behavior recommendations (e.g., handwashing) that may not be directly related to COVID-19 because it is airborne [38]. See the Supplementary Materials for measures included in these analyses.

### 2.3. Demographics

Participants provided demographic information that included, among others, age, birth sex, and racial and ethnic identity. Given the predominately White sample, a binary race/ethnicity variable was created in which White = 0 and all other racial identities and/or Hispanic ethnicity = 1.

### 2.4. Impulsivity

In Sample 1, a modified version of three subscales (sensation seeking, positive urgency, negative urgency) from the UPPS-P Impulsive Behavior Scale [39] was used to measure impulsivity during Wave 2 of data collection. In an effort to reduce participant burden, only these three subscales from the UPPS-P were included and the six items with the highest factor loadings from each of the original subscales were retained. Cronbach’s alphas for the three subscales were as follows: α = 0.81, α = 0.83, and α = 0.79, respectively. In Sample 2, impulsivity was measured with the Short UPPS-P Impulsive Behavior Scale (Short UPPS-P [40]) assessing the same three subscales (four items per subscale) for consistency across samples. For this sample, alpha values were 0.70 (sensation seeking), 0.76 (positive urgency), and 0.73 (negative urgency). Both samples completed Likert-like items ranging from 1 (“strongly agree”) to 4 (“strongly disagree”) with higher values corresponding to greater levels of impulsivity.

### 2.5. Affect and Anxiety

The GAD-7 [41] is a seven-item measure used to assess feelings of generalized anxiety over the past two weeks (e.g., “feeling nervous, anxious, or on edge”). Items are rated on a 0 (“not at all”) to 3 (“nearly every day”) scale and summed (α = 0.92), with higher scores corresponding to greater levels of anxiety. Additionally, the Positive and Negative Affect Schedule for Children (PANAS-C [42]) was used to assess positive (e.g., pride) and negative (e.g., sadness) affective states over the past two weeks. Items were rated on a 1 (“very slightly or not at all”) to 5 (“extremely”) scale. Items within each factor were then summed with higher values indicating greater positive (α = 0.89) or negative (α = 0.84) affect.

### 2.6. COVID-19 Safety Measure Norms, Perceived Effectiveness, and Behaviors

Two items were used to assess descriptive norms. Specifically, individuals were asked about their perceptions of others’ (people their own age who live in the town where they are residing) engagement in indoor (i.e., wearing a face mask in public indoor places like grocery stores, retail stores, etc.) and outdoor (i.e., wearing a face mask in outdoor public places like parks or busy sidewalks) mask use. An additional two items were used to assess how effective respondents believed indoor and outdoor mask use to be. Items were rated on a 1 (“no one,” “not at all effective”) to 5 (“everyone,” “extremely effective”) scale. Similarly, to assess personal engagement in COVID-19 safety behaviors, individuals were asked how often they engaged in indoor and outdoor mask use. Items were rated on a 1 (“never”") to 5 (“always”) scale. Individuals also reported on how often they engaged in social distancing from 0 (“never”) to 10 (“always”). Social distancing was defined as remaining away from settings where one would gather with others, avoiding mass gatherings, and maintaining distance of approximately 6 feet from others whenever possible, outside of people with whom you live.

### 2.7. News Exposure

Time spent watching the news and time spent searching the news were indexed separately to account for passive versus active exposure to COVID-19-related news. Exposure to COVID-19-related news was indexed using two items: “I watch a lot of news about the coronavirus (COVID-19)” and “I spend a huge percentage of my time trying to find updates online or on TV about coronavirus.” Items were rated on a 1 (“not true at all for me”) to 7 (“very true for me”) scale.

### 2.8. State Response Timing

Individuals identified their state of current residence to determine the impact of state response timing to COVID-19 on compliance with safety behaviors. Reponses were then dichotomized into early response (i.e., stay-at-home mandate or advisory before 1 April 2020) and late response (i.e., stay-at-home mandate or advisory on 1 April 2020 or later/never) states [16].

### 2.9. Data Analysis

Data were analyzed in SAS version 9.4 [43] and Mplus version 7.31 [44] using a series of linear and ordinal regressions. More specifically, three outcomes (social distancing and each type of mask use) were regressed onto 18 demographic, trait, affective, social (e.g., norms), and contextual (e.g., news exposure) variables in 48 (3 outcomes ∗ 18 independent variables) separate models to assess the role of each of these factors on compliance with COVID-19 safety measures. All regression models were conducted controlling for race/ethnicity, sex, age, and state response timing. Given the number of inferential tests conducted, a false discovery rate procedure was applied [45,46]. This approach controls for false positives by testing the probability that the null is true given the null has been rejected. Following the procedure developed by Benjamini and Hochberg [45], *p*-values were sorted into ascending order for each of the 48 inferential tests. Next, adjusted *p*-values were calculated using d ∗ (i/n), where d = the false discovery rate (0.05), i = *p*-value rank (from 1 to 48), and n = the total number of regressions run (48). A main effect was considered significant if the observed *p*-value was less than or equal to the adjusted *p*-value. Effect sizes are presented as *R^2^* in which small, medium, and large effects are defined as *R^2^* values of 0.01, 0.09, and 0.25, respectively [47]. In addition to the regression models, differences in norms, perceived effectiveness, and behaviors as a function of state response timing were assessed using independent sample *t*-tests.

## 3. Results

### 3.1. Non-COVID-19-Specific Indicators of Compliance

Means and correlations among all variables are presented in Table 1. The results of regression analyses examining main effects are presented in Table 2. Identifying as White, compared to non-White, was associated with decreased engagement in outdoor mask use (β = −0.16, R^2^ = 0.03, *p* < 0.01), but race/ethnicity was not significantly associated with indoor mask use or social distancing. Females were more likely to engage in indoor mask use than males (β = 0.33, R^2^ = 0.11, *p* < 0.001), but no differences were found for social distancing or outdoor mask use. There were no significant effects of age on any outcome. Greater anxiety was associated with greater social distancing and indoor and outdoor mask use (βs = 0.11–0.14; R^2^s = 0.01, *p*s = 0.02). Greater positive affect was related to less social distancing compliance, while greater negative affect was associated with greater compliance (β = −0.17, R^2^ = 0.03, *p* = < 0.001; β = 0.12, R^2^ = 0.01, *p* < 0.01, respectively). Negative affect was also positively associated with outdoor mask use (β = 0.13, R^2^ = 0.02, *p* < 0.01). However, both positive and negative affect were not related to indoor mask use. With regard to impulsivity, no facets (i.e., positive urgency, negative urgency, and sensation seeking) exhibited significant relations with any outcome in the full sample. Models examining relations between impulsivity and subsequent outcomes were also conducted separately for Sample 2 to see if the results differed, given that for Sample 1, impulsivity data were collected at Wave 2, whereas norms and perceived effectiveness were collected at Wave 3 (nearly 3 years later). Three significant main effects emerged in Sample 2 after controlling for multiple comparisons using a false discovery rate analysis. Significant main effects emerged between sensation seeking and both social distancing (β = −0.17; R^2^ = 0.03, *p* < 0.01) and indoor mask use (β = −0.30; R^2^ = 0.09, *p* < 0.001). Additionally, positive urgency was significantly related to indoor mask use (β = −0.28; R^2^ = 0.08, *p* < 0.001).

### 3.2. COVID-19-Specific Indicators of Compliance

Descriptive norms for indoor mask use were significantly related to indoor mask use (β = 0.41, R^2^ = 0.17, *p* < 0.001; see Table 2). No relations emerged for indoor mask descriptive norms on social distancing or outdoor mask use. Descriptive norms for outdoor mask use were associated with social distancing (β = 0.12, R^2^ = 0.01, *p* < 0.01), indoor mask use (β = 0.23, R^2^ = 0.05, *p* < 0.01), and outdoor mask use (β =0.49, R^2^ = 0.24, *p* < 0.001). Indoor and outdoor mask perceived effectiveness were significantly related to all outcomes (βs = 0.29–0.60, R^2^s = 0.08–0.37, *p*s < 0.001). Both time spent watching and time spent searching for COVID-19 news were positively related to social distancing and outdoor mask use (βs = 0.16–0.25, R^2^s = 0.03–0.06, *p*s < 0.001) but not indoor mask use. Delayed state response timing was significantly related to all outcomes (βs = −0.15–−0.31, R^2^s = 0.02–0.10, *p*s < 0.01). Importantly, looking at state response timing, independent *t*-tests found that those residing in early response states endorsed higher levels of social distancing, indoor mask use, perceived effectiveness of all behaviors, and descriptive norms (see Table 3). 

## 4. Discussion

COVID-19 has had a global impact across a myriad of facets of life including physical and mental health, economics, and education. Several behavioral recommendations have been made to help reduce the spread of COVID-19 and mitigate subsequent adverse outcomes. Recent data have suggested that young adults in particular are at increased risk of spreading COVID-19 [1]. As such, the current work aimed to evaluate who is likely to engage in social distancing and indoor and outdoor mask use among current and former undergraduate college students across the U.S. Key findings from this study highlight the importance of cognitive and contextual factors including norms, perceived effectiveness, news exposure, and state response timing as mechanisms for increasing safety behavior compliance.

### 4.1. Demographic Indicators of Compliance

Consistent with extant literature on demographic indicators of COVID-19 safety behaviors [19,25], men in this sample were less likely to use masks when indoors than women and this relation evinced a medium effect size. Several potential explanations for this finding have been posited with research suggesting that men are more likely than women to view mask wearing as an infringement on their independence [48] and that women are more likely to be receptive to data-driven resources and feel a greater social responsibility than men to engage in these behaviors [35]. Examination of ethnic/racial differences found that White individuals were less likely to engage in outdoor mask use compared to non-White individuals. This is contrary to previous findings of Czeisler et al. [18], who found racial differences in approval of safety behaviors, but no differences in actual engagement in these behaviors. One potential explanation for these discrepant results is that, as data presented to the public showed that racial minority individuals have higher morbidity rates following infection due to systemic inequality (e.g., access to medical care), racial minority individuals may have become more likely to take steps to minimize the potential for infection than their White counterparts. Additionally, as noted above, White individuals endorse lower approval of safety behaviors [18], so it could be that the impact of lower approval of safety precautions on behavior has become more influential over the course of the pandemic, creating ethnic/racial differences with regard to compliance. Interestingly, White individuals are more likely to express intention to get vaccinated against COVID-19 than their Black and Hispanic counterparts [49]. However, among those that reported no intention to receive a vaccine, Black and Hispanic individuals were more likely to report refusal due to systemic barriers (e.g., lack of time) and mistrust regarding the vaccine as compared to their White counterparts. These discrepancies may be routed in historical medical mistreatment of racial minorities. Intention to receive a vaccine among racial minority groups could be improved by programs offering paid leave and transportation to obtain vaccines and by reaching out to trusted community leaders to help address minority community’s vaccine mistrust.

### 4.2. Affect, Impulsivity, and Compliance

With regard to affect and anxiety, the current study yielded small-to-medium effect sizes suggesting that greater anxiety and negative affect are related to increased compliance with less regulated safety behaviors (i.e., social distancing, outdoor mask use). These findings support extant literature that notes positive relations between stress and/or anxiety and compliance with COVID-19 safety behaviors [20,21]. Given the cross-sectional nature of the data, it is not discernable whether increased levels of negative affect and anxiety lead to increased compliance, if greater compliance increases anxiety/negative affect (e.g., following guidelines decreases time spent with others that increases negative affect), or whether a third variable (e.g., presence of a chronic health condition, employment changes) explains the relation between these variables. Given that positive affect is negatively associated with social distancing and negative affect is positively associated, it may be that individuals who are less compliant are experiencing decreased disruption to their routines, whereas those who are more compliant are experiencing more disruption and subsequently more negative affect and anxiety.

Broadly, we anticipated that greater impulsivity, particularly sensation seeking, would predict decreased compliance, but this hypothesis was not supported in the full sample. Three significant relations emerged between impulsivity facets and compliance for Sample 2 (i.e., the sample in which impulsivity was assessed at the same timepoint as compliance) after adjusting for multiple comparisons. Notably, in this sample, greater positive urgency was associated with decreased compliance with indoor mask use and sensation seeking was associated with decreased social distancing and indoor mask use compliance. It could be the significant relations between impulsivity and compliance were hidden in the full sample due to a three-year gap between assessment of impulsivity and compliance in Sample 1. As demonstrated, there were lower norms and perceived effectiveness ratings in the delayed response group and, as such, impulsivity may have exacted a larger effect on this group than in early response states with higher levels of norms and perceived effectiveness. Future work should aim to further discern relations between impulsivity and safety behavior compliance. Additionally, assessing under what contexts individuals are likely to comply with mask use (e.g., at the store vs. the bar/restaurant; alone vs. with others) would enable public health officials to gain a more nuanced understanding of the relations between impulsivity and compliance.

### 4.3. Descriptive Norms, Perceived Effectiveness, and Compliance

Indoor mask descriptive norms exhibited a medium effect on indoor mask usage but not on social distancing or outdoor mask use, whereas outdoor mask descriptive norms were related to all outcomes with effect sizes ranging from small to large. These discrepancies could be due to the fact that indoor mask use is more widely regulated than outdoor mask use (e.g., masks required to enter stores) and, thus, perceptions of indoor mask use are less impactful given the limited degree of choice compared to outdoor mask use. Though it is clear that peer norms influence behavior, it is important to note that perceived norms do not have to be accurate to be influential, and that individuals tend to underestimate the percentage of peers engaging in health promotive behaviors [50]. Strategies to increase accurate perceptions of peer engagement in safety behaviors, such as mask use, social distancing, and vaccination, could increase personal intention to engage in these behaviors [12]. Like norms for outdoor mask use, perceived effectiveness seems to influence norms both within and across behaviors (e.g., mask norms on social distancing), with greater perceived effectiveness of indoor and outdoor mask use relating to greater compliance with all outcomes. Notably, relations between perceived effectiveness on mask use yielded medium-to-large effect sizes (*R^2^*s = 0.14–0.37) suggesting strong relations. As such, informational campaigns aimed at increasing feelings of perceived efficacy of one safety behavior could lead to greater compliance with additional behaviors. Further, access to information about the effectiveness of COVID-19 health promotion behaviors could serve to increase perceived effectiveness and compliance.

### 4.4. News Exposure and Compliance

Both time spent watching and searching for COVID-19-related news resulted in small-to-medium significant relations with social distancing and outdoor mask use but not indoor mask use. Given that many states and businesses required the use of masks inside public spaces, the lack of relationship between news exposure and indoor mask use could be due to limited choice in indoor mask use. However, when behaviors are less regulated, such as with social distancing and outdoor mask use, interaction with related news seemed to impact behavior engagement. Exposure to news itself, however, is likely not sufficient for increasing COVID-19 safety compliance; we must consider the accuracy of the news that is being presented. Notably, the present study found positive associations between news exposure and compliance, but the accuracy of the information or perceptions about the news being consumed was not assessed. It is plausible that given our sample is primarily an educated sample, they may be more likely to seek out news sources that promote compliance with safety behaviors [51]. However, misinformation regarding the pandemic has been far too common and exposure to inaccurate news could lead to decreased compliance with safety behaviors, ranging from mask use to intention to receive a COVID-19 vaccination [52]. In fact, Simonov et al. [53] demonstrated how influential news exposure can be on COVID-19 safety behavior engagement. Specifically, the authors found that increased viewership of Fox News decreased social distancing and stay-at-home compliance, and that for every 10.0% increase in Fox News viewership, stay-at-home adherence decreased by 8.9%. Further, these authors calculated the persuasion rate (a measure of the influence of viewership on behavior accounting for baseline behavior) of Fox News exposure on stay-at-home compliance from March 2020 to April 2020 and found persuasion rates ranging from 27.7% (when controlling for zip code trends) to 710.0% (when the sample was restricted to television service subscribers), suggesting that Fox News has had a high propensity to negatively influence COVID-19 stay-at-home behaviors amongst viewers. This highlights the importance of understanding what makes news messaging an effective tool for behavior compliance (or non-compliance).

In their review on social interventions to assist with mitigating the spread of COVID-19, Van Bavel et al. [52] highlight that information must come from trusted individuals or sources and that messaging is most effective when it appeals to individual’s morals or norms. Further, this review discussed several studies demonstrating that increased trust in health officials led to increased likelihood of compliance with social distancing and Ebola safety measures [52]. Importantly, the timing of government response could potentially shape the messaging presented in these states and vice versa. For example, states with an early response to the pandemic may also have received more prosocial messaging about mask use, which in turn influences perceived effectiveness, norms, and personal compliance. Mixed messaging between state and federal officials could create confusion about the effectiveness of safety measures; as such, it is crucial for officials at all levels of government to be consistent in their messaging. Additionally, the type of messaging used impacts compliance. For example, individuals were more likely to self-isolate when presented with prosocial messaging that aimed to elicit a positive emotional response than when presented with threatening messages [54]. Taken together, utilization of trusted community leaders to provide prosocial messaging related to COVID-19 tailored to their populations could serve to increase compliance at the community level and, in turn, at the national and global levels. 

### 4.5. Timing of Responses to Health Crises

The present findings highlight the importance of state response timing of stay-at-home orders on subsequent social distancing and mask use behaviors, norms, and perceived effectiveness. These findings have several implications for government response to health crises. It appears that early intervention at the government level has not only immediate consequences including reductions in new infections, hospitalizations, and deaths [55], but also long-term consequences (e.g., compliance with safety behaviors that could slow the spread of disease). This is consistent with extant findings among German citizens that found that mandatory mask mandates resulted in compliance rates of 96%, compared to only 77% compliance when masks were voluntary [6]. Importantly, mask compliance predicted greater compliance with additional behaviors such as hand washing and social distancing. These findings may call for a greater federal response to health pandemics as a means for creating compliance and ultimately slowing the spread of the pandemic. For example, New Zealand implemented federally mandated stay-at-home, mask, and travel orders at the outset of the pandemic and, as a result, was able to quell the spread of the pandemic early [56]. Further, and perhaps of interest to elected officials, individuals perceived mask mandates as more fair than voluntary policies, suggesting that governments may benefit from greater health safety compliance and more favorable evaluations of health policies (e.g., mask use, stay-at-home orders) when implementing mandates as opposed to advisories.

Importantly, though state response timing was indicative of compliance, norms, and perceived effectiveness, it is likely that there are bidirectional relations among these variables. For example, state leaders may have felt pressure to delay stay-at-home mandates in response to negative attitudes from their constituents regarding these actions. Further, individuals are likely to elect officials with similar ideologies and, thus, attitudes and behaviors regarding pandemic responses may precede actions by state officials. Additionally, a third variable such as political affiliation could explain relations between response timing and COVID-19 safety measure perceived effectiveness, norms, and behaviors [57,58,59]. This speculation again highlights that a consistent response at the federal level may have a greater likelihood of increasing compliance and slowing the spread of pandemics as opposed to the state-by-state implementation of safety measures [60].

### 4.6. Future Directions and Limitations

As noted in the methods, COVID-19-related data were collected in November 2020 for Sample 1 and from January to February 2021 for Sample 2. In the U.S., vaccines became available in December 2020, with those designated at-risk or essential workers having earliest access to the vaccine. Though we did not assess vaccination status or plans, it is likely the large majority of our young adult sample were not vaccinated at the time of assessment. As such, it will be important to assess how the present findings may change in response to vaccine availability and vaccination status. However, the findings presented here are important to understanding initial behavioral responses to public health crises when vaccine access is extremely limited.

Though the present work covered a wide range of indicators of compliance, limitations do exist. Notably, these data are cross-sectional and, thus, causal relations among variables cannot be determined. As with much self-reported behavioral research, responses may be biased due to difficulty with recall and method bias [56]. Further, though participants were informed that responses were anonymous, participants may have over-reported how frequently they engage in safety behaviors in order to appear more pro-social [61]. Additionally, our sample was comprised of current and former college students and, as such, results may not generalize to emerging adults outside of formal educational settings or to other age groups. Participants were recruited from four universities and self-selected into the study; thus, results may not generalize to other college samples. Further, due to sample size limitations, all non-White participants were grouped together, limiting our ability to gain a more nuanced understanding of the impact of racial identity on compliance. In addition, data came from two different samples that were assessed a few months apart. With regard to specific measures used, two subscales of impulsivity within the UPPS-P, lack of planning and lack of premeditation, were removed to reduce participant burden and, thus, were not available for analysis. Further, for Sample 1, UPPS-P survey data were collected at Wave 2, whereas COVID-19 norms, perceived effectiveness, and behaviors were collected at Wave 3 (almost 3 years later), and though the UPPS-P was developed to assess trait-level impulsivity, impulsivity can change over time [62]. Importantly, we conducted supplemental analyses examining the associations with UPPS-P within Sample 2 alone and the results differed such that three significant relations emerged after controlling for multiple comparisons. Additionally, although social distancing was an outcome of interest, norms and perceived effectiveness regarding social distancing were not assessed. Lastly, the current study used the PANAS-C, a measure that has only been validated through age 18, and thus may not generalize to all study participants as ages for the participants at time of data collection ranged from 19 to 27 years. However, the items in PANAS-C are very similar to the full PANAS. Specifically, the items used in the child scale to measure positive (e.g., lively) and negative (e.g., afraid) affect are identical or nearly identical to items from short-form PANAS measures of positive (e.g., active) and negative (e.g., afraid) affect that are not specifically designed for children [63]. Moreover, evidence suggests a similar factor structure of the full PANAS across children, adolescents, and young adults [64]. As such, use of the PANAS-C likely does not significantly detract from the validity of the present findings.

## 5. Conclusions

This study aimed to add to extant literature by describing how demographic, social, and contextual influences (such as norms, attitudes, news exposure, and state response timing) impact health behavior engagement among current and former college students. The present results suggest that increasing feelings of perceived effectiveness and modifying descriptive norms are two crucial means for increasing individual compliance, and that campaigns targeting compliance should consider attitudes and norms as key tools in their messaging. Affective indicators and impulsivity are largely not indicative of compliance, suggesting that demographic (e.g., race, sex) and contextual indicators (e.g., perceived effectiveness, norms, news exposure) are more closely related to compliance. Findings are important for broader public health as they highlight the impact of early government response to global health crises. They also speak to the importance of consistent access to accurate information as a means to increasing individual engagement with health promoting behaviors.

## Figures and Tables

**Table 1:** Means (M) and correlations among demographic, individual, and COVID-19 indicators.

	M	1	2	3	4	5	6	7	8	9	10	11	12	13	14	15	16	17	18	19
1. Age	21.95	-																		
2. Race/Ethnicity	0.70	−0.07	-																	
3. Sex	0.73	**0.10**	0.00	-																
4. State Response Timing	0.43	0.08	0.08	**0.12**	-															
5. GAD-7	8.35	0.04	**−0.06**	**0.18**	**0.11**	-														
6. PANAS-C Negative	11.06	0.02	0.02	0.07	0.07	**0.58**	-													
7. PANAS-C Positive	13.74	−0.02	**0.18**	0.00	**0.28**	**−0.31**	**−0.33**	-												
8. Negative Urgency	8.68	0.04	0.02	0.01	0.08	**0.26**	**0.22**	−0.04	-											
9. Positive Urgency	7.24	0.02	−0.09	**−0.15**	0.06	**0.13**	**0.11**	−0.02	**0.62**	-										
10. Sensation Seeking	10.31	0.01	0.01	**−0.19**	0.00	-0.01	0.02	**0.15**	**0.19**	**0.40**	-									
11. COVID-19 News Watch	3.36	0.00	0.01	**0.14**	0.02	**0.13**	**0.10**	-0.01	0.04	0.04	0.05	-								
12. COVID-19 News Search	2.47	0.02	−0.01	0.09	−0.05	**0.18**	**0.14**	−0.06	0.08	**0.10**	0.05	**0.73**	-							
13. Indoor Mask Norms	40.02	−0.01	−0.03	0.02	**−0.31**	−0.03	0.00	−0.07	−0.05	−0.05	0.02	−0.02	−0.03	-						
14. Outdoor Mask Norms	30.02	0.04	**−0.16**	−0.05	**−0.37**	**−0.10**	−0.06	**−0.15**	−0.08	0.02	0.07	0.03	0.09	**0.48**	-					
15. Indoor Mask Perceived Effectiveness	4.67	0.02	**−0.12**	**0.10**	**−0.25**	0.03	0.02	**−0.10**	−0.08	−0.09	−0.07	**0.14**	**0.16**	**0.21**	**0.21**	-				
16. Outdoor Mask Perceived Effectiveness	4.36	0.05	**−0.20**	0.03	**−0.28**	0.06	0.05	**−0.12**	−0.04	−0.03	−0.05	**0.17**	**0.22**	**0.13**	**0.31**	**0.66**	-			
17. Social Distancing Behavior	6.68	0.00	**−0.11**	0.07	**−0.15**	**0.10**	**0.11**	**−0.21**	−0.07	−0.05	**−0.11**	**0.19**	**0.17**	0.05	**0.15**	**0.33**	**0.34**	-		
18. Indoor Mask Behavior	4.74	−0.05	−0.03	**0.28**	**−0.23**	**0.18**	0.07	**−0.12**	−0.09	**−0.23**	**−0.18**	**0.11**	**0.11**	**0.47**	**0.27**	**0.50**	**0.40**	**0.42**	-	
19. Outdoor Mask Behavior	3.71	0.01	**−0.19**	0.03	**−0.31**	0.09	**0.11**	**−0.20**	**−0.12**	−0.08	−0.05	**0.24**	**0.28**	**0.16**	**0.55**	**0.45**	**0.65**	**0.48**	**0.56**	**-**

N = 490. Variables were coded such that non-White = 0 and White = 1; male = 0 and female = 1; early state response timing = 0 and late state response timing = 1. Norms, perceived effectiveness, and mask behaviors were coded on a 1 to 5 scale with higher values corresponding to greater norms, perceived effectiveness, and compliance. Social distancing was coded on a 1 to 10 scale with higher values corresponding to greater compliance. Bold indicates items significantly correlated at *p* < 0.05.

**Table 2:** Main effects for COVID-19 behavior engagement as a function of demographics, affect, impulsivity, news exposure, norms, and perceived behavior effectiveness.

Indicator.	Social Distancing		Indoor Mask Use		Outdoor Mask Use	
	β	*R^2^*	β	*R^2^*	β	*R^2^*
Covariate Indicators						
Age	−0.01	0.00	−0.05	0.00	0.01	0.00
Race/ethnicity	−0.10	0.01	−0.02	0.00	−0.16 **	0.03
Sex	0.08	0.01	0.33 ***	0.11	0.07	0.00
State Response Timing	−0.15 **	0.02	−0.26 ***	0.07	−0.31 ***	0.10
Covariate Model		0.04		0.16		0.13
Affect and Impulsivity Indicators						
GAD-7	0.11 *	0.01	0.14	0.02	0.11*	0.01
PANAS-C Negative	0.12 **	0.01	0.06	0.00	0.13 **	0.02
PANAS-C Positive	−0.17 ***	0.03	−0.05	0.00	−0.08	0.01
Negative Urgency	−0.06	0.00	−0.04	0.00	−0.09	0.01
Positive Urgency	−0.04	0.00	−0.16 *	0.03	−0.07	0.00
Sensation Seeking	−0.10	0.01	−0.09	0.01	−0.03	0.00
COVID-19 Indicators						
COVID News Watch	0.19 ***	0.04	0.05	0.00	0.23 ***	0.05
COVID News Search	0.16 ***	0.03	0.06	0.00	0.25 ***	0.06
Indoor Mask Norms	−0.01	0.00	0.41 ***	0.17	0.07	0.00
Outdoor Mask Norms	0.12 **	0.01	0.23 **	0.05	0.49 ***	0.24
Indoor Mask Perceived Effectiveness	0.29 ***	0.08	0.45 ***	0.20	0.38 ***	0.14
Outdoor Mask Perceived Effectiveness	0.31 ***	0.10	0.38 ***	0.14	0.61 ***	0.37

Variables were coded such that non-White = 0 and White = 1; male = 0 and female = 1; early state response timing = 0 and late state response timing = 1. Early response is defined as a stay-at-home order before 1 April 2020. Late response is defined as a stay-at-home order on 1 April 2020 or later, including states that never implemented an order. Each main effect model was conducted individually (N = 476–490). Models reported in this table controlled for age, race, sex, and response timing. Given the number of models tested, a false discovery rate correction was applied to determine significance. Significant models after applying the correction are denoted as follows: * = *p* < 0.05, ** = *p* < 0.01, *** = *p* < 0.001. Analyses were also conducted using the PROC MIXED procedures in SAS for multilevel modeling with university as the nesting variable to account for random variance that may be attributable to university affiliates. Results were generally consistent with the linear regression models presented in Table 2, and no significant interactions emerged between university affiliation and the independent variables. Using multilevel modeling, state response timing was no longer a significant indicator of any outcome despite moderate to large Betas (βs = −0.59 to 0.80). Additionally, multilevel models examining relations between state response timing and subsequent outcomes resulted in large standard errors (values ranged from 0.48 to 0.49). This is likely due to the fact 206 of 209 participants in late response states were affiliated with the same university. As such, there is multicollinearity between the university affiliation and state response timing that is masking the significance of this variable and resulting in inflated standard errors. Given that models were largely unchanged and the present issues with multicollinearity between university and state response timing, the results derived from linear regressions are presented).

**Table 3:** Means and standard deviations of COVID-19 safety protocol behavior engagement, perceived norms, and perceived effectiveness by state response timing.

	Early Response M (SD)	Late Response M (SD)
Social Distancing Behavior	70.01 (2.34)	6.24 (2.84)
Indoor Mask Behavior	4.83 (0.53)	4.61 (0.87)
Outdoor Mask Behavior	40.07 (1.13)	3.23 (1.48)
Indoor Mask Norms	4.25 (0.53)	3.71 (0.87)
Outdoor Mask Norms	3.33 (0.91)	2.59 (0.94)
Indoor Mask Perceived Effectiveness	4.79 (0.44)	4.50 (0.70)
Outdoor Mask Perceived Effectiveness	4.54 (0.63)	4.13 (0.78)

N = 476–490. Using independent sample *t*-tests, all means significantly differed by response timing at *p* < 0.01. Early response is defined as a stay-at-home order before 1 April 2020. Late response is defined as a stay-at-home order on 1 April 2020 or later, including states that never implemented an order.

## Data Availability

Not applicable.

## Supplementary Materials

The following are available online at https://www.mdpi.com/article/10.3390/ijerph18168715/s1, Supplementary File S1: Assessment Battery.
