# Supplementary material for: Individual and COVID-19-Specific Indicators of Compliance with Mask Use and Social Distancing: The Importance of Norms, Perceived Effectiveness, and State Response
Source: Int J Environ Res Public Health. 2021 Aug 18;18(16):8715. doi: 10.3390/ijerph18168715 (PMC8394116; doi:10.3390/ijerph18168715)
Supplement: Supplementary file 1 [file ijerph-18-08715-s001.zip › ijerph-1252774-supplementary.pdf]

Supplemental File: Assessment Battery

Appendix A – Overview of Measures

Appendix B: Demographics (e.g., age, sex, race, year in school, major, marital status).

American College Personnel Association (2013, December). ACPA standards for demographics questions. Retrieved from http://www.myacpa.org/sites/default/files/Proposal-Demographic-Questions-and-Responses-2.pdf

The GenIUSS Group (2014). *Best practices for asking questions to identify transgender and other gender minority respondents on population-based surveys.* J. L. Herman (Ed.) Los Angeles, CA: The Williams Institute.

Appendix C: Short UPPS-P Impulsive Behavior Scale:

Cyders, M. A., Littlefield, A. K., Coffey, S., & Karyadi, K. A. (2014). Examination of a short English version of the UPPS-P Impulsive Behavior Scale. *Addictive Behaviors*, *39*(9), 1372-1376.

Appendix D: UPPS-P Impulsive Behavior Scale:

Lynam, D. R., Smith, G. T., Whiteside, S. P., & Cyders, M. A. The UPPS-P: Assessing five personality pathways to impulsive behavior. West Lafayette, IN: Purdue University, 2006, 10

Appendix E: Positive and Negative Affect Scale (PANAS)

Watson, D., Clark, L. A., & Tellegen, A. (1988). Development and validation of brief measures of positive and negative affect: the PANAS scales. *Journal of personality and social psychology*, *54*(6), 1063-1070.

Appendix F: Generalized Anxiety Disorder 7-item (GAD-7) Scale

Spitzer, R. L., Kroenke, K., Williams, J. B., & Löwe, B. (2006). A brief measure for assessing generalized anxiety disorder: the GAD-7. *Archives of internal medicine*, *166*(10), 1092-1097.

Appendix G: COVID-19 News Items

COVID-19 Exposure, Perceptions, and Media Usage Questions adapted from the PhenX Toolkit

Appendix H: COVID-19 Items

The CoRonavIruS Health Impact Survey (CRISIS) V0.3: Adult Self-Report Form

The CoRonavIruS Health Impact Survey (CRISIS) V0.3. Adult Self-Report Follow Up Form: Current Form: National Institute of Mental Health (NMIH); [Available from: https://www.nlm.nih.gov/dr2/CRISIS_Adult_Self-Report_Follow_Up_Current_Form_V0.3.pdf.

Appendix B: Demographic Questionnaire

Q434 What is your current student/employment status

- ◯ Full-time undergraduate student taking all classes remotely (4)
- ◯ Full-time undergraduate student taking all classes in-person (5)
- ◯ Full-time undergraduate student taking a hybrid of remote and in-person classes (6)
- ◯ Part-time undergraduate student taking all classes remotely (7)
- ◯ Part-time undergraduate student taking all classes in-person (8)
- ◯ Part-time undergraduate student taking a hybrid of remote and in-person classes (9)
- ◯ Graduate student taking all classes remotely (10)
- ◯ Graduate student taking all classes in-person (11)
- ◯ Graduate student taking a hybrid of remote and in-person classes (12)
- ◯ Working full-time, not taking classes (13)
- ◯ Working part-time, not taking classes (14)
- ◯ Other (15) ____

C2Res Where did you currently live

- ○ Campus housing (1)
- ○ Apartment/house with parent(s) (2)
- ○ Apartment/house with friend's family (3)
- ○ Apartment/house with friend(s)/roommate(s)/partner (4)
- ○ Apartment/house where I live alone (5)
- ○ Other: please specify (6) ____

C1LivingW Who did you live with (check all that apply)

- ▢ Parent(s) (1)
- ▢ Sibling(s) (2)
- ▢ Other family member(s) (3)
- ▢ Friend(s) (4)
- ▢ Significant other (5)
- ▢ No one (6)
- ▢ Other (7) ____

C2State What state did you currently live in

▼ Alabama (1) ... I do not reside in the United States (53)

Page Break

C2Age How old are you

- 19 (19)
- 20 (20)
- 21 (21)
- 22 (22)
- 23 (23)
- 24 (24)
- 25 (25)
- 26 or older (26)

Q436 Are you of Hispanic or Latinx origin?

- No (1)
- Yes (2)

Q437 What race/ethnicity do you consider yourself (select all that apply)?

- ▢ American Indian or Alaska Native (1)
- ▢ Asian (2)
- ▢ Black or African American (3)
- ▢ Native Hawaiian or other Pacific Islander (4)
- ▢ White (5)
- ▢ Other (6)

Q438 What sex were you assigned at birth, on your original birth certificate

- ○ Male (1)
- ○ Female (2)

C2Gender What is your gender identity (check all that apply)

- ▢ Male (1)
- ▢ Female (2)
- ▢ Trans male/Trans man (3)
- ▢ Trans female/Trans woman (4)
- ▢ Gender queer/Gender non-conforming (5)
- ▢ Different identity (please state) (6) ____

Q439 What is your sexual orientation?

- ○ Straight (1)
- ○ Gay or lesbian (2)
- ○ Bisexual (3)
- ○ Transgender, transsexual, or gender non-conforming (4)

Q440 Student status

- ○ Full-time (12+ credits) (1)
- ○ Part-time (1-11 credits) (2)

Q441 Dating/marital status

- Divorced/separated (1)
- Married (2)
- Living with a partner (3)
- Committed relationship (4)
- Casual dating relationship (5)
- single (6)

Q442 Are you a student athlete

- No (1)
- Yes, varsity sport (2)
- Yes, club sport or intramural sport (3)

Appendix C: Short UPPS-P

Q449 INSTRUCTIONS: Below are a number of statements that describe ways in which people act and think. For each statement, please indicate how much you agree or disagree with the statement. Be sure to indicate your agreement for every statement below.

	Strongly Agee (1)	Agree Somewhat (2)	Disagree Somewhat (3)	Strongly Disagree (4)
I generally like to see things through to the end. (1)				
My thinking is usually careful and purposeful. (2)				
When I am in a great mood I tend to get into situations that could cause me problems. (3)				
Unfinished tasks really bother me. (4)				
I like to stop and think things over behavior I do them. (5)				
When I feel bad, I will often do thinks I later regret in order to make myself feel better now. (6)				
Once I get going on something I hate to stop. (7)				
Sometimes when I feel bad, I can't seem to stop what I am doing even though it is making me feel worse. (8)				
I quite enjoy taking risks. (9)				

I tend to lose control when I am in a great mood. (10)	○	○	○	○
I finish what I start. (11)	○	○	○	○
I tend to value and follow a rational, “sensible” approach to things. (12)	○	○	○	○
When I am upset I often act without thinking. (13)	○	○	○	○
I welcome new and exciting experiences and sensations, even if they are a little frightening and unconventional. (14)	○	○	○	○
When I feel rejected, I will often say things that I later regret. (15)	○	○	○	○
I would like to learn to fly an airplane. (16)	○	○	○	○
Others are shocked or worried about the things I do when I am feeling very excited. (17)	○	○	○	○
I would enjoy the sensation of skiing very fast down a high mountain slope. (18)	○	○	○	○
I usually think carefully before doing anything. (19)	○	○	○	○

I tend to act without thinking when I am really excited. (20)	○	○	○	○

Appendix D: UPPS-P

Below are a number of statements that describe ways in which people act and think. For each statement, please indicate how much you agree or disagree with the statement. If you **Agree Strongly** circle **1**, if you **Agree Somewhat** circle **2**, if you **Disagree somewhat** circle **3**, and if you **Disagree Strongly** circle **4**. Be sure to indicate your agreement or disagreement for every statement below. Also, there are questions on the following pages.

	**Agree Strongly**	**Agree Some**	**Disagree Some**	**Disagree Strongly**
3. I generally seek new and exciting experiences and sensations.	1	2	3	4
12. I often get involved in things I later wish I could get out of.	1	2	3	4
17. When I feel bad, I will often do things I later regret in order to make myself feel better now.	1	2	3	4
22. Sometimes when I feel bad, I can't seem to stop what I am doing even though it is making me feel worse.	1	2	3	4
23. I quite enjoy taking risks.	1	2	3	4
25. When I am really ecstatic, I tend to get out of control.	1	2	3	4
29. When I am upset I often act without thinking.	1	2	3	4
31. I welcome new and exciting experiences and sensations, even if they are a little frightening and unconventional.	1	2	3	4
34. When I feel rejected, I will often say things that I later regret.	1	2	3	4
35. Others are shocked or worried about the things I do when I am feeling very excited.	1	2	3	4
36. I would like to learn to fly an airplane.	1	2	3	4
44. I often make matters worse because I act without thinking when I am upset.	1	2	3	4
50. In the heat of an argument, I will often say things that I later regret.	1	2	3	4
52. I tend to act without thinking when I am really excited.	1	2	3	4
54. When I am really happy, I often find myself in situations that I normally wouldn't be comfortable with.	1	2	3	4
57. When I am very happy, I feel like it is ok to give in to cravings or overindulge.	1	2	3	4
58. Sometimes I do impulsive things that I later regret.	1	2	3	4
59. I am surprised at the things I do while in a great mood.	1	2	3	4

Appendix E: Positive and Negative Affect Scale for Children (PANAS-C)

This scale consists of a number of words that describe different feelings and emotions. Indicate to what extent you have felt this way **during the past two weeks**

	Very slightly or not at all (1)	A little (2)	Moderately (3)	Quite a bit (4)	Extremely (5)
Sad (Sad)	○	○	○	○	○
Upset (Upset)	○	○	○	○	○
Scared (Scare)	○	○	○	○	○
Miserable (Miserable)	○	○	○	○	○
Cheerful (Cheerful)	○	○	○	○	○
Proud (Proud)	○	○	○	○	○
Joyful (Joyful)	○	○	○	○	○
Lonely (Lonely)	○	○	○	○	○
Delighted (Delighted)	○	○	○	○	○
Lively (Lively)	○	○	○	○	○

Appendix F: GAD-7

C2GAD7 **Over the last 2 weeks**, how often have you been bothered by the following problems?

	Not at all (0)	Several days (1)	Over half the days (2)	Nearly every day (3)
Feeling nervous, anxious, or on edge (Nervous)	○	○	○	○
Not being able to stop or control worrying (Worrying)	○	○	○	○
Worrying too much about different things (Worry alot)	○	○	○	○
Trouble relaxing (No relax)	○	○	○	○
Being so restless that it's hard to sit still (Restless)	○	○	○	○
Becoming easily annoyed or irritable (Annoyed)	○	○	○	○
Feeling afraid as if something awful might happen (Afraid)	○	○	○	○

Appendix G: COVID-19 News Items

C2COVIDNews

	Not at all true for me (1)	(2)	(3)	(4)	(5)	(6)	Very true for me (7)
I watch a lot of news about the coronavirus (COVID-19). (C1COVIDNews_12)	○	○	○	○	○	○	○
I spend a huge percentage of my time trying to find updates online or on TV about coronavirus (C1COVIDNews_13)	○	○	○	○	○	○	○

Appendix H: COVID-19 Items

C2SocDis In the **last 2 weeks,** how much have you been social distancing?

- ○ Not at all (1)
- ○ (2)
- ○ (3)
- ○ (4)
- ○ (5)
- ○ (6)
- ○ (7)
- ○ (8)
- ○ (9)
- ○ At all times (10)

C2PercepBehav1

	What % of other people your age in the town/state you currently live engage in this behavior					How frequently do you engage in this behavior						How effective do you believe this behavior is for keeping you safe from COVID-19				
	Everyone	Many people	Some people	Few people	No one	All the time	Often	Sometimes	Once in a While	Never	N/A	Very effective	Somewhat effective	Neither effective or ineffective	Somewhat ineffective	Not at all effective
Wear a face mask in public indoor places like grocery stores, retail stores, etc. (C2MaskIn)	○	○	○	○			○	○	○	○		○	○	○	○	○
Wear a face mask in outdoor public places like parks or busy sidewalks (C2MaskOutCrowd)	○	○	○	○			○	○	○	○		○	○	○	○	○
